# Characterization of the Complete Chloroplast Genome of *Hygroryza aristata* (Retz.) Nees ex Wight & Arn. (Zizaniinae, Poaceae)

**DOI:** 10.1080/23802359.2021.1935352

**Published:** 2021-06-22

**Authors:** Haoqi Wang, Zhiqiang Wu, Guohua Fan, Gang Zheng, Cuihua Gu, Luke R. Tembrock, Zuoren Yang, Jiankang Yang, Jie Wang

**Affiliations:** a Zhengzhou Research Base, State Key Laboratory of Cotton Biology, Zhengzhou University, Zhengzhou, China; b Shenzhen Branch, Guangdong Laboratory for Lingnan Modern Agriculture, Genome Analysis Laboratory of the Ministry of Agriculture, Agricultural Genomics Institute at Shenzhen, Chinese Academy of Agricultural Sciences, Shenzhen, China; c Shanxi Agricultural University/Shanxi Academy of Agricultural Sciences, Sorghum Research Institute. Yuci, Shanxi, China; d School of Landscape and Architecture, Zhejiang Provincial Key Laboratory of Germplasm Innovation and Utilization for Garden Plants, Key Laboratory of National Forestry and Grassland Administration on Germplasm Innovation and Utilization for Southern Garden Plants, Zhejiang A & F University, Hangzhou, China; e Department of Agricultural Biology, Colorado State University, Fort Collins, CO, USA; f State Key Laboratory of Cotton Biology, Institute of Cotton Research, Chinese Academy of Agricultural Sciences, Anyang, China; g School of Basic Medical Sciences, Dali University, Dali, China

**Keywords:** Crop wild relatives, Poaceae, plastome, phylogeny, aquatic plants

## Abstract

The complete chloroplast genome sequence of *Hygroryza aristata* was sequenced, assembled and published for the first time here. The chloroplast genome was 135,681 bp in length and comprised of a large single-copy (LSC, 81,532 bp) region and a small single-copy (SSC, 12,383 bp) region interspersed by two inverted repeats (IRs, 20,883 bp). Gene annotation resulted in the identification of 113 unique genes including 79 protein-coding genes, 30 transfer RNA (tRNA) genes, and four ribosomal RNA (rRNA) genes. In addition, 118 simple sequence repeats (SSRs) and 47 long repeats were identified. Phylogenetic analysis based on maximum likelihood analysis (ML) resolved the placement of *H. aristata* sister to a clade of *Rhynchoryza subulata* and *Zizania*.

*Hygroryza aristata* (Retz.) Nees ex Wight & Arn. (Zizaniinae, Poaceae) is a perennial, aquatic, stoloniferous grass that forms extensive floating mats in ponds and lakes. The species is distributed across Southern China and Southeast Asia including Bangladesh, Cambodia, India, Laos, Malaysia, Myanmar, Nepal, Pakistan, Sri Lanka, Thailand, and Vietnam (Morya et al. 2017). This species is planted in artificial waterscapes, used as food for poultry, and a source of chemical compounds like lignan and indole alkaloids which are used medicinally for anti-inflammatory and antioxidant properties (Chung et al. 2011). Herein, we characterized the complete chloroplast genome of *H. aristata* for the first time and performed phylogenetic analysis to resolve the relationship among close relatives, which will provide a genomic resource for studying Poaceae evolution and breeding markers for rice wild relatives.

Fresh leaves of *H. aristata* were sampled from greenhouse grown plants at the Institute of Botany of the Chinese Academy of Sciences in Beijing (Beijing, China; 39°54′20″ N, 116°25′29″ E) and stored for later use with accession code HA20190314C (Specimen Museum of Botany of the Chinese Academy of Sciences in Beijing, ZQ Wu, wuzhiqiang@caas.cn). The total cellular DNA was extracted using the cetyltrimethyl ammonium bromide (CTAB) method (Doyle 1987) and purified with phenol extraction (Yang et al. 2014). PCR amplification and Sanger sequencing methods were employed to obtain the whole chloroplast genome of *H. aristata*. The entire chloroplast was sequenced in overlapping fragments from PCR products using the chloroplast primers from Wu et al. (2009). The PCR amplicons were purified following Tang et al. (2010) and directly sequenced on an ABI 3730 (Applied Biosystems, Foster City, CA, USA). The final Sanger sequences were trimmed and assembled with the ContigExpress program from the Vector NTI Suite 6.0 (Informax Inc., North Bethesda, MD). The final assembled chloroplast sequence was submitted to DOGMA v1.2 (Wyman et al. 2004) for annotation using *Zizania aquatica* as reference. The final annotation was submitted to GenBank and given the accession MW849262. Simple sequence repeats (SSRs) across the chloroplast genome were identified using MISA (Beier et al. 2017) searching for motif sizes from one to six nucleotide units with minimum thresholds set to 8, 5, 4, 3, 3, and 3 repeat units for mono-, di-, tri-, tetra-, penta-, and hexa-nucleotide SSRs, respectively. REPuter (Kurtz et al. 2001) was employed to detect long repeats across the chloroplast genome with default settings.

Similar to most typical angiosperms, the chloroplast genome of *H. aristata* was 135,681 bp in size and divided into a large single-copy (LSC, 81,532 bp) region and a small single-copy (SSC, 12,383 bp) region separated by a pair of inverted repeats (IRs, 20,883 bp). The overall GC content was 39.03%, and 37.17%, 33.44%, 44.32% in the LSC, SSC, and IRs, respectively. A total of 113 unique genes were identified (17 of which were duplicated in the IRs) consisting of 79 protein-coding genes, 30 transfer RNA (tRNA) genes, and four ribosomal RNA (rRNA) genes. From this, 16 genes were found to contain intron(s) including eight tRNA genes and eight protein coding genes. As for the SSR identification, there were 108 mono-nucleotide, 4 di-nucleotide, 2 tri-nucleotide, and 4 tetra-nucleotide repeats totaling 118 SSR loci. Additionally, 34 forward matches and 13 palindromic matches were recognized from the long repeat analyses.

To investigate the phylogenetic position of the *H. aristata* chloroplast, we constructed a phylogenetic tree based on sampling from 23 Oryzeae species chloroplast genome sequences published in NCBI, and *Phyllostachys propinqua* (Arundinariinae) chloroplast genome was selected as an outgroup. Alignment was accomplished in MAFFT v7 (Katoh et al. 2019). The best-fit substitution model for the data was Blosum62 + F + I + G4 as chosen in ModelFinder v1.6.8 (Kalyaanamoorthy et al. 2017). The maximum likelihood tree was obtained in IQ-Tree v1.6 (Nguyen et al. 2015) with 1,000 bootstrap replicates. As shown in Figure 1, H*. aristata* was most closely related to a clade of *Rhynchoryza subulata* and *Zizania*.

**Figure 1.**
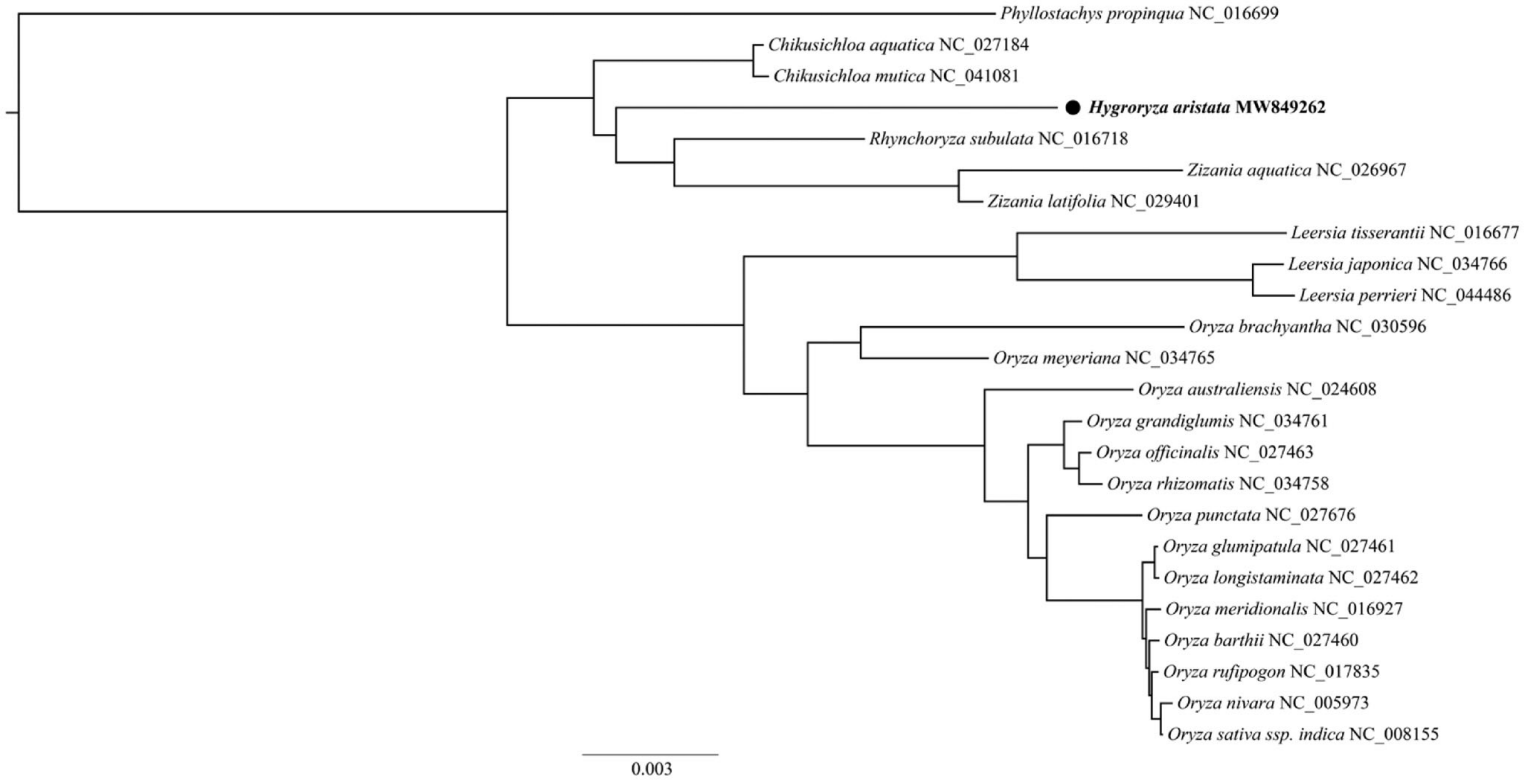
A maximum likelihood tree indicating the position of *H. aristata* with other chloroplast sequences from Oryzeae species and *Phyllostachys propinqua* (Arundinariinae) as an outgroup. All nodes were fully supported (Bootstrap value = 100).

## Data Availability

The data that support the findings of this study are openly available in GenBank at https://www.ncbi.nlm.nih.gov/genbank/, MW849262.
